# Short-term replacement of starch with isomaltulose enhances both insulin-dependent and -independent glucose uptake in rat skeletal muscle

**DOI:** 10.3164/jcbn.17-98

**Published:** 2018-03-17

**Authors:** Keiichi Koshinaka, Rie Ando, Akiko Sato

**Affiliations:** 1 Department of Health and Sports, Niigata University of Health and Welfare, 1398 Shimami-cho, Kita-ku, Niigata 950-3198, Japan

**Keywords:** isomaltulose, rat, insulin, exercise, AMPK

## Abstract

Dietary intervention for preventing postprandial increases in glucose level by replacing high-glycemic index (GI) carbohydrates with lower-GI carbohydrate has been proposed as a strategy for treating insulin-resistant metabolic disorders such as type II diabetes. In this study, we examined the effect of short-term replacement of starch with a low-GI disaccharide, isomaltulose, on insulin action in skeletal muscle. Male Wistar rats were fed isomaltulose for 12 h during their dark cycle. In isolated epitrochlearis muscle, insulin-induced glucose uptake was greater in tissue from rats treated with isomaltulose than from those treated with starch. This insulin-sensitizing effect occurred independently of changes visceral fat mass. To determine whether this sensitization was specific to insulin stimulation, we also measured glucose uptake in response to exercise. In isolated epitrochlearis muscles from rats that performed swimming exercise, exercise-induced glucose uptake was higher in isomaltulose-treated than starch-treated animals. This amplification was associated with increased phosphorylation of exercise-induced AMP-activated protein kinase. In conclusion, our results demonstrate that short-term replacement of starch with isomaltulose enhances both insulin-dependent and -independent glucose uptake in isolated skeletal muscle. This transient replacement of carbohydrate with isomaltulose, together with exercise, represents a potentially effective approach for the management of insulin resistance.

## Introduction

Sustained postprandial hyperglycemia is a risk factor for development of insulin resistance.^(1,2)^ The glycemic responses of various dietary carbohydrates are influenced by multiple factors, including not only the amount of carbohydrate but also the type. Glycemic index (GI) is a relative ranking of carbohydrates and carbohydrate-containing foods based on how quickly they affect blood glucose level. A growing body of evidence suggests that consumption of high-GI carbohydrates, such as rapidly digestible sucrose, and foods containing them, may increase the risk of insulin resistance, whereas consumption of lower-GI carbohydrates and foods may counteract this detrimental effect.^(1,2)^ Thus, dietary intervention aimed at preventing increases in postprandial glucose level by replacing high-GI carbohydrate with lower-GI carbohydrate has been proposed as a strategy for treating insulin-resistant metabolic disorders such as type II diabetes.

Isomaltulose (6-*O*-d-glucopyranosyl-d-fructose; ISO), an isomer of sucrose, is a naturally occurring disaccharide consisting of glucose and fructose linked by α-1,6-glucosidic bond.^(3)^ Like sucrose, isomaltulose is completely digestible and has 4 kcal/g caloric value, but this stable linkage considerably reduces the rate of digestion relative to that of sucrose, which has α-1,2-glucosidic bond. Consequently, ingested ISO is absorbed slowly, resulting in a very low GI 32.^(4)^ Previous studies in human subjects and rodents have demonstrated that chronic treatment with ISO, replacing high-GI carbohydrates, decreases fasting insulin and improves peripheral insulin action,^(5–8)^ as judged by homeostasis model assessment, euglycemic hyperinsulinemic clamp, and oral glucose tolerance tests.^(5,8–10)^ These results indicate that chronic use of ISO as an alternative to high-GI carbohydrates may have beneficial effects on the management of glucose homeostasis in insulin-resistant subjects. Because skeletal muscle is main site for glucose disposal when glucose loading is performed in the postprandial state, muscle is a likely site of improvement of peripheral insulin action following ISO treatment. To date, however, no direct evidence demonstrating the insulin-sensitizing effect of ISO on skeletal muscle has been obtained. Therefore, in this study, we focused on skeletal muscle to investigate the connection between ISO and skeletal muscle and elucidate the molecular mechanisms of this effect.

In chronic ISO treatment, one positive factor contributing to muscle insulin action is reduction in visceral fat mass. Indeed, several studies have demonstrated that chronic ISO treatment attenuates accumulation of visceral fat.^(6–8,10)^ However, ISO treatment has a positive effect on peripheral insulin action even in non-obese and non-insulin-resistant subjects,^(5)^ as well as in insulin-resistant rats, without reduction of visceral fat mass,^(9)^ indicating that ISO treatment could have an impact on muscle insulin action independently of reduction in visceral fat mass as observed after chronic ISO treatment (several weeks or more). Accordingly, we hypothesized here that short-term ISO treatment, in the absence of changes in fat mass, could enhance muscle insulin action, and that ISO might exert this effect via responses occurred in skeletal muscle itself, rather than through secondary effects in other tissues. This study was performed to test this hypothesis.

To identify the effect of ISO on skeletal muscle per see and simplify analysis of its molecular mechanisms, we employed an *ex vivo* system using skeletal muscle isolated from rats treated with ISO. Under specific conditions in which the effect insulin action on glucose uptake is sensitized, sensitivity of glucose uptake to insulin-independent stimuli is also amplified through an insulin-independent mechanism.^(11)^ Hence, to further characterize the effect of ISO on muscle insulin action, we also investigated whether the expected insulin-sensitizing effect of ISO was specific to insulin stimulation. Because exercise is well known to be an inducer of the muscle glucose uptake and an effective strategy for managing insulin resistance, we used a physical exercise task as an insulin-independent stimulus.

## Materials and Methods

### Animals

This research was approved by the Animal Studies Committee of Niigata University of Health and Welfare. Male Wistar rats were obtained from CLEA Japan (Tokyo, Japan). Rats were housed individually at constant room temperature (23 ± 1°C) in a 12-h light (06:00–18:00)/12-h dark cycle, and were provided standard laboratory chow and water *ad libitum*.

### Experimental diets

The compositions of experimental diets are shown in Table 1. The diets were isocaloric (3.9 kcal/g) but contained different types of carbohydrates. As percent of calories, diets supplied 27% as protein, 12% as fat and 61% as carbohydrate. All materials, except ISO (Mitsui Sugar, Tokyo, Japan), were obtained from Oriental Yeast (Tokyo, Japan).

### Experimental design

Rats weighing ~130 g were assigned randomly to either a starch *ad libitum*, starch pair-fed, sucrose *ad libitum*, or ISO *ad libitum* group. Just before the dark cycle (18:00), the food was changed to experimental diets containing the indicated sugars, and the rats had access to the food container until the end of the dark cycle (06:00). During the dark cycle, locomotor activity was continuously measured (Supermex; Muromachi Kikai, Tokyo, Japan). After 3-h fasting (09:00), some rats underwent exercise consisting of eight 20-s bouts of swimming while loaded with a weight equal to 18% of body weight, with 40 s of rest between bouts.^(12–14)^ These exercised and non-exercised rats in a comparison were accustomed to swimming for 10 min/day, 2 days before the experiment. Rats were anesthetized with by intraperitoneal injection of sodium pentobarbital (50 mg/kg body weight) or volatile isoflurane (MK-A110D; Muromachi Kikai) according to the manufacturer’s instructions, followed by dissection of the epitrochlearis muscles. Some of these muscles were clamp-frozen in liquid nitrogen for measurement of muscle metabolites [glycogen and triglyceride (TG)] and Western blot analysis, and the remaining samples were used for subsequent incubation, as described below. After muscle dissection was completed, retroperitoneal and epididymal fat-pads were removed and weighted.

### Muscle incubation

The muscles were incubated with shaking for 20 min at 30°C in 4 ml of oxygenated Krebs-Henseleit buffer (KHB) containing 40 mM mannitol and 0.1% radioimmunoassay-grade bovine serum albumin (BSA), in the absence or presence of purified human insulin (100 µU or 10 mU/ml). Flasks were gassed continuously with 95% O_2_-5% CO_2_ during incubation. After incubation, some muscles were clamp-frozen in liquid nitrogen for Western blot analysis, and the remaining samples were used for subsequent measurement of glucose uptake.

### Muscle incubation for hypoxia stimulation

To study the effect of hypoxia on glucose uptake, muscles were incubated for 80 min at 35°C in hypoxygenated KHB containing 8 mM glucose, 32 mM mannitol, and 0.1% BSA under hypoxic condition (95% N_2_-5% CO_2_).^(15)^ Thereafter, muscles were incubated for 15 min at 30°C in oxygenated KHB (95% O_2_-5% CO_2_) containing 40 mM mannitol and 0.1% BSA, and used for subsequent measurement of glucose uptake.

### Glucose uptake *in vitro*

2-Deoxyglucose (2DG) was used to measure the rate of muscle glucose uptake according to a previously described method.^(16)^ After 20-min or 15-min incubation, as described above, muscles were further incubated for 20 min at 30°C in 4 ml of oxygenated KHB containing 8 mmol/L 2DG, 32 mmol/L mannitol, and 0.1% BSA without or with insulin at the same concentration as in the initial 20-min incubation (described above). The flasks were gassed continuously with 95% O_2_-5% CO_2_ during incubation. After incubation, the muscles were blotted dry and clamp-frozen in liquid nitrogen for fluorometric measurement of 2DG-6-phosphate (2DG6P).

### Glycogen recovery after exercise

At 09:00, rats consuming the indicated experimental diets during the dark cycle underwent exercise as described above. Immediately after exercise and 2 h after exercise (11:00), rats received glucose solution (1.5 mg/kg body weight) orally to facilitate glycogen recovery. Four h after exercise (13:00), epitrochlearis muscles were dissected out under anesthesia, and then clamp-frozen in liquid nitrogen for measurement of glycogen.

### Muscle metabolites

Muscles were homogenized in 0.3 M perchloric acid (PCA), and the extracts were used to measure glycogen by the amyloglucosidase method.^(17)^ The remaining PCA extracts were centrifuged at 1,000 *g* for 10 min at 4°C. After centrifugation, the supernatant was collected and neutralized by the addition of 2M KOH, followed by fluorometric measurements of 2DG6P.^(18)^ For TG measurement, muscles were homogenized in water with 5% Triton X-100, heated for 3 min at 90°C, and then cooled with ice. After one more heating/cooling cycle, samples were centrifuged at 1,000 *g* for 10 min at 4°C. The resultant supernatants were used in assay with the TG kit (Sigma-Aldrich, St. Louis, MO).

### Blood parameters

Blood glucose was measured using the product Glutest Every (Sanwa Kagaku Kenkyusho, Nagoya, Japan). Plasma insulin concentrations were measured using enzyme immunoassay kits (Morinaga Institute of Biological Science, Yokohama, Japan). Plasma levels of TG and free fatty acid (FFA) were determined enzymatically using the TG kit (Sigma-Aldrich) and NEFA-c test kit (Wako Pure Chemical Industries, Osaka, Japan), respectively.

### Immunoblot analysis

Muscles were homogenized in ice-cold buffer containing 50 mM HEPES (pH 7.4), 150 mM NaCl, 10% glycerol, 1% Triton X-100, 1.5 mM MgCl_2_, 1 mM EDTA, 10 mM Na_4_P_2_O_7_, 100 mM NaF, 2 mM Na_3_VO_4_, 2 mM PMSF, aprotinin (10 µg/ml), and leupeptin (10 µg/ml).^(13)^ The homogenates were rotated end-over-end at 4°C for 60 min, and then centrifuged at 10,000 *g* for 10 min at 4°C. Aliquots of supernatants were used for immunoblot analysis. Briefly, supernatants were electrophoretically separated by SDS-PAGE and transferred to PVDF membranes. The membranes were incubated overnight at 4°C with antibodies against AMP-activated protein kinase (AMPK) α, p-AMPK Thr^172^, Akt, p-Akt Thr^308^, p-TBC1D1 Ser^237^, TBC1D4, p-TBC1D4 Thr^642^, p-TBC1D4 Ser^588^, or glucose transporter (GLUT) 4, followed by incubation for 90 min with HRP-conjugated anti–rabbit IgG. Antibody against GLUT4 was obtained from Biogenesis (Poole, UK), and antibodies against p-TBC1D1 Ser^237^ and TBC1D4 were obtained from Millipore (Temecula, CA), respectively. Other antibodies were from Cell Signaling Technology (Beverly, MA). Immunoreactive bands were visualized using enhanced chemiluminescence reagent (GE Healthcare Japan, Hino, Japan), and quantified using NIH Image.

### Statistics

Values are expressed as means ± SE. Differences among multiple groups were determined using a one-way analysis of variance (ANOVA) followed by the Tukey-Kramer test. When two mean values were compared, analysis was performed using an unpaired *t* test. *P*<0.05 was considered significant.

## Results

**Fig. 1:**
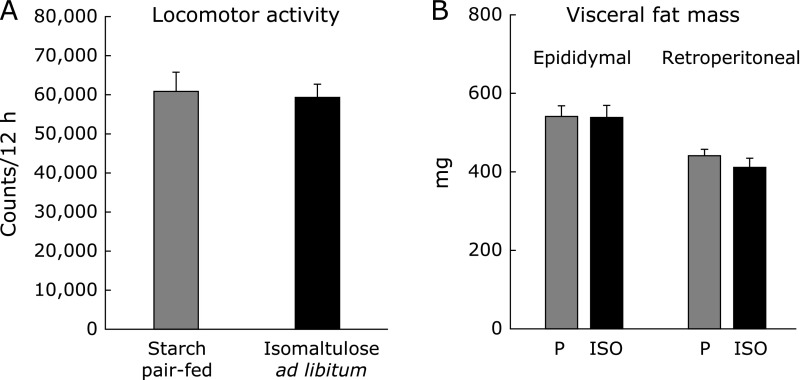
Locomotor activity (A) was measured during the dark cycle (12-h). After 3-h fasting following the end of dark cycle, visceral (epididymal and retroperitoneal) fat was dissected out, and was weighed (B). P: starch pair-fed, ISO: isomaltulose *ad libitum*. Values are means ± SE (*n* = 6).

During the dark cycle, we measured locomotor activity, a positive regulator for muscle insulin action (Fig. 1A). Compared with starch pair-feeding, ISO treatment did not affect the activity level. As expected, visceral fat mass, as measured from the epididymal and retroperitoneal regions, was similar in both groups (Fig. 1B), validating the experimental model as enabling comparison between groups with equal levels of visceral fat mass.

After 3-h fasting following the end of the dark cycle, blood parameters were measured (Table 2). Starch pair-feeding significantly decreased circulating glucose and TG levels, and increased FFA and glycerol levels in comparison to starch *ad libitum*. By contrast, ISO treatment did not exert significant effects on these parameters, except TG, in comparison to starch *ad libitum*, and consequently these parameters significantly differed relative to the starch pair-fed group. ISO treatment resulted in further reduction in triglyceride level compared to starch pair-feeding. Insulin levels were comparable among groups.

**Fig. 2:**
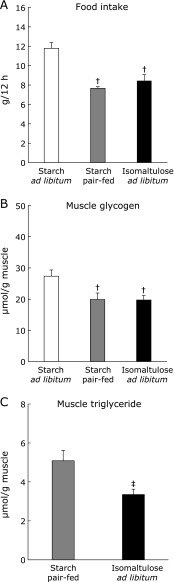
Food intake (A) was measured during the dark cycle (12-h). After 3-h fasting, muscles were dissected out, and were used for measurement of muscle glycogen (B) and triglyceride contents (C). Values are means ± SE (*n* = 6–9). ^†^*p*<0.05 vs starch *ad libitum* group. ^‡^*p*<0.05 vs starch pair-fed group.

As shown in Fig. 2A, the amount of food intake was roughly one-third lower in the starch pair-fed group than in the starch *ad libitum* group, but identical to that in the ISO group. As a reflection of food intake, starch pair-feeding decreased muscle glycogen to the same extent as ISO treatment, relative to starch *ad libitum* (Fig. 2B). By contrast, muscle TG was significantly lower in the ISO treatment group than in the starch pair-fed group (Fig. 2C).

**Fig. 3:**
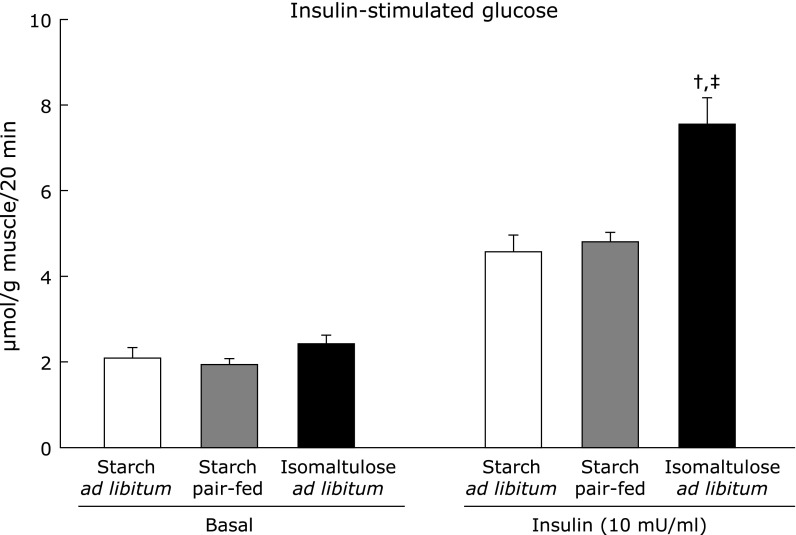
Insulin-stimulated glucose uptake. After 3-h fasting following the end of dark cycle, muscles were dissected out, and then were incubated for 20 min in KHB in the absence (basal) or presence of insulin (10 mU/ml). After the incubation, glucose uptake was measured in KHB with 2-deoxyglucose in the same insulin concentrations which used in the preceding incubation. Values are means ± SE (*n* = 5–6). ^†^*p*<0.05 vs starch *ad libitum* group. ^‡^*p*<0.05 vs starch pair-fed group.

Under these metabolic conditions, glucose uptake was measured in isolated epitrochlearis muscle (Fig. 3). There was no difference in basal glucose uptake among groups. In response to the maximally effective concentration of insulin (10 mU/ml), glucose uptake in starch *ad libitum* and starch pair-fed groups was increased similarly, to 2.2- and 2.5-fold respectively, whereas a 3.1-fold increase was observed in ISO *ad libitum* group, significantly larger than in the other groups.

**Fig. 4:**
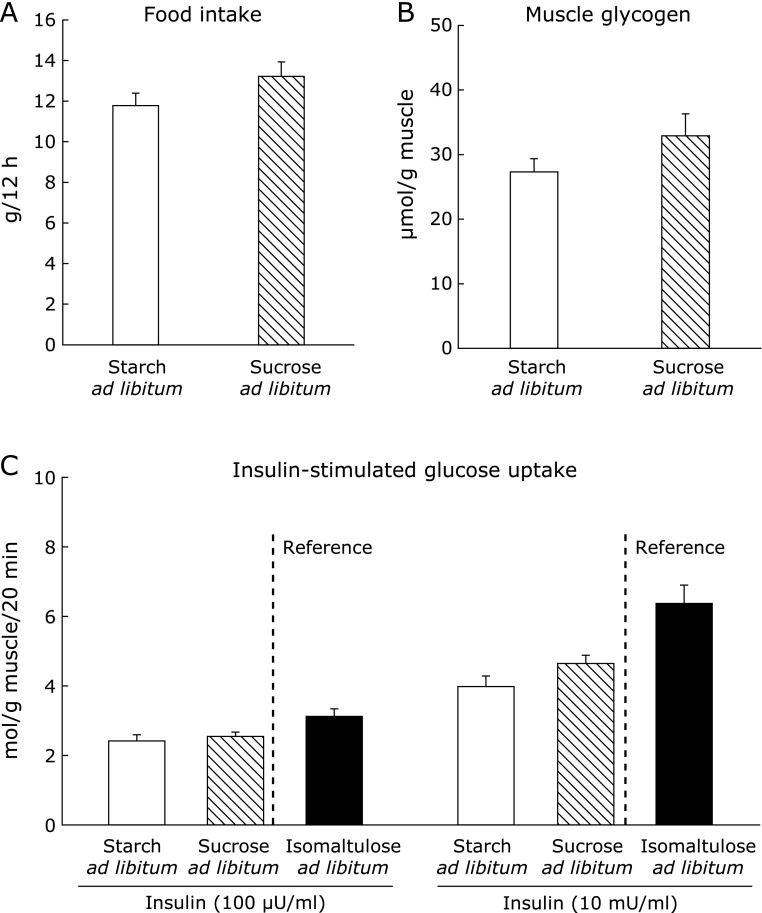
Effect of sucrose feeding. Food intake (A) was measured during the dark cycle (12-h). After 3-h fasting, muscles were dissected out, and were used for measurement of muscle glycogen content (B). Some isolated muscles were incubated for 20 min in KHB in the absence (basal) or presence of insulin (100 µU or 10 mU/ml). After the incubation, glucose uptake was measured in KHB with 2-deoxyglucose in the same insulin concentrations which used in the preceding incubation (C). The values of ISO *ad libitum* group were shown as a positive control. Values are means ± SE (*n* = 5–6).

To investigate the possibility that the insulin-sensitizing effect of ISO observed in the comparison between starch and ISO was due to the different forms of carbohydrate (disaccharide vs polysaccharide), we examined the effect of sucrose, another disaccharide, as an alternative to ISO. During the dark cycle, food intake in rats eating sucrose was comparable to that in rats eating starch (Fig. 4A). Therefore, we did not perform a pair-feeding experiment in this comparison. Sucrose feeding resulted in similar muscle glycogen levels (Fig. 4B). Under the same levels of food intake and glycogen, we also measured glucose uptake in isolated epitrochlearis muscle (Fig. 4C). When muscles were stimulated with physiological (100 µU/ml) and supra-physiological (10 mU/ml) levels of insulin, glucose uptake did not differ between muscles of starch-fed and sucrose-fed rats (Fig. 4C). This result indicates that the enhanced insulin action observed in ISO feeding was not due to the difference in carbohydrate form.

**Fig. 5:**
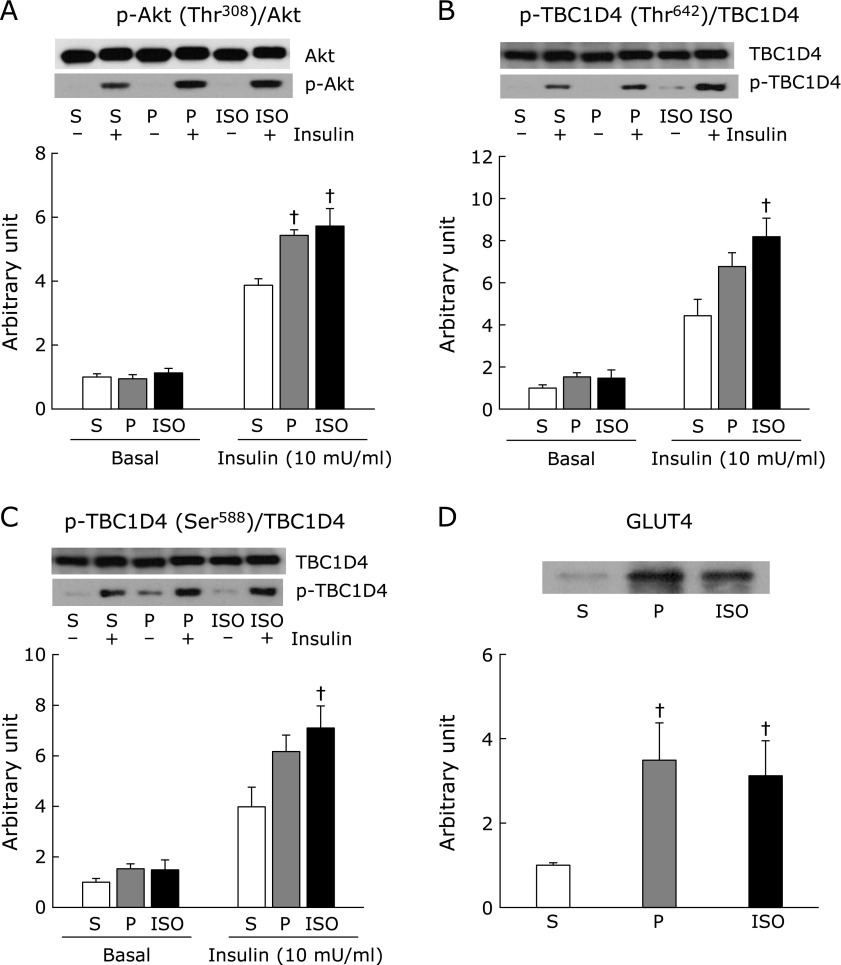
After 3-h fasting following the end of dark cycle, muscles were dissected out, and then were incubated for 20 min in KHB in the absence (basal) or presence of insulin (10 mU/ml). Western blot analysis was conducted with antibodies against p-Akt (Thr^308^) and Akt (A), p-TBC1D4 (Thr^642^) and TBC1D4 (B), p-TBC1D4 (Ser^588^) and TBC1D4 (C), and glucose transporter 4 (D). A representative blot of phosphoprotein (lower panel) and total protein (upper panel) is shown above each figures, and blot intensity is expressed as phosphoprotein/total protein (A, B, C). S: starch *ad libitum*, P: starch pair-fed, ISO: isomaltulose *ad libitum*. Values are means ± SE (*n* = 6). ^†^*p*<0.05 vs starch *ad libitum* group.

In skeletal muscle, insulin induces translocation of GLUT4 from the intracellular pool to the plasma membrane to facilitate uptake of glucose. Activation of phosphatidylinositol-3 kinase (PI3K) is essential for insulin-stimulated GLUT4 translocation. Insulin activates PI3K, leading to phosphorylation and activation of Akt, followed by subsequent phosphorylation of TBCID4, one of the most distal signaling proteins involved in inducing insulin-stimulated GLUT4 translocation.^(19,20)^ When isolated epitrochlearis muscles were stimulated by insulin, marked increases in phosphorylation level were observed in p-Akt Thr^308^ (Fig. 5A), p-TBC1D4 Thr^642^ (Fig. 5B), and p-TBC1D4 Ser^588^ (Fig. 5C) relative to the basal levels of the corresponding proteins. Phosphorylation levels in these molecules tended to be higher in the starch pair-feeding group than the starch *ad libitum* group, but the difference reached statistical significance level only in p-Akt (Thr^308^) (Fig. 5A). ISO feeding induced significantly higher phosphorylation levels in all of these molecules in comparison with starch *ad libitum* feeding, but these levels were not different from those observed in starch pair-feeding. There was no difference among groups in basal phosphorylation levels of these molecules. Also, total proteins of Akt and TBC1D4 were not different among groups (Akt; 1.00 ± 0.04, 0.98 ± 0.03, 1.00 ± 0.02, TBC1D4; 1.00 ± 0.09, 0.91 ± 0.09, 0.86 ± 0.13 in Starch *ad libitum*, Starch pair-fed, ISO *ad libitum*, respectively). Total GLUT4 protein levels in starch pair-fed and ISO *ad libitum* groups were similar, and significantly higher than in the starch *ad libitum* group (Fig. 5D).

**Fig. 6:**
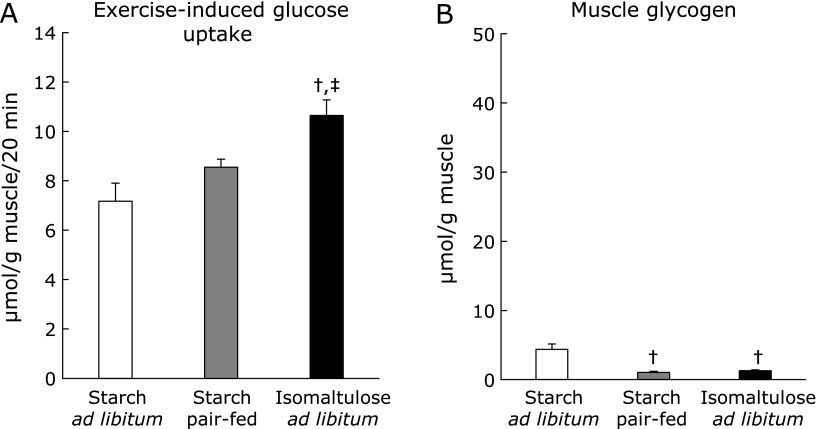
Exercise-induced glucose uptake. After 3-h fasting following the end of dark cycle, rats underwent swimming exercise. Immediately after the exercise, muscles were dissected out, and then were incubated for 20 min in KHB. After the incubation, glucose uptake was measured in KHB with 2-deoxyglucose (A). Some muscles were used for measurement of muscle glycogen content (B). Values are means ± SE (*n* = 5–6). ^†^*p*<0.05 vs starch *ad libitum* group. ^‡^*p*<0.05 vs starch pair-fed group.

To obtain further insight, we also investigated whether the glucose uptake–promoting effect of ISO was specific to insulin stimulation. Physical exercise is well characterized as an inducer of muscle glucose uptake. Like insulin stimulation, exercise and exercise-related stimuli (e.g., electrically induced muscle contraction and hypoxia) initiate GLUT4 translocation, but independently of PI3K activation.^(19–21)^ Therefore, we measured the effect of ISO feeding on insulin-independent exercise-induced glucose uptake (Fig. 6A). Immediately after exercise, glucose uptake was significantly elevated in all groups. The levels were comparable between starch *ad libitum* and Starch pair-fed groups, whereas a higher level was observed in the ISO *ad libitum* group than in the other groups (Fig. 6A). Exercise consumed large amounts of glycogen, to extent of depletion (Fig. 6B). Compared to the starch *ad libitum* group, lower levels of glycogen were observed in both starch pair-fed and ISO *ad libitum* groups, with no difference between groups (Fig. 6B).

**Fig. 7:**
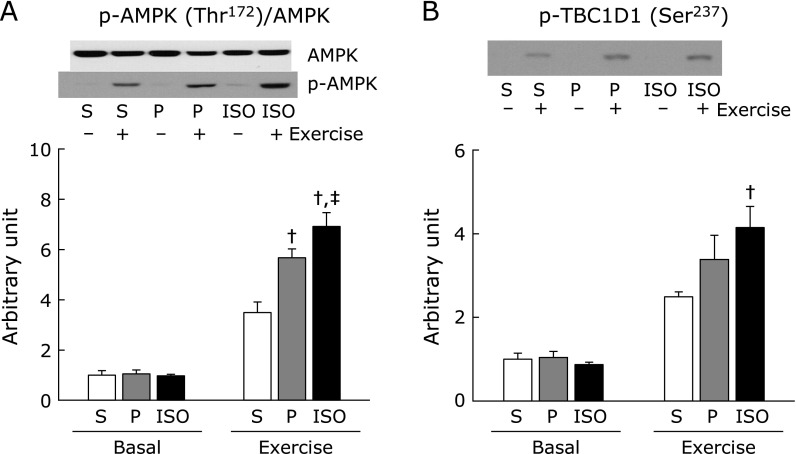
After 3-h fasting following the end of dark cycle, rats underwent swimming exercise. Immediately after the exercise, muscles (including muscles from non-exercised rats) were dissected out, and were used for Western blot analysis with antibodies against p-AMPK (Thr^172^) and AMPK (A), and p-TBC1D1 (Ser^237^) (B). A representative blot of phosphoprotein (lower panel) (A, B) and total protein (upper panel) (A) is shown above each figures, and blot intensity is expressed as phosphoprotein/total protein (A). S: starch *ad libitum*, P: starch pair-fed, ISO: isomaltulose *ad libitum*. Values are means ± SE (*n* = 6). ^†^*p*<0.05 vs starch *ad libitum* group. ^‡^*p*<0.05 vs starch pair-fed group.

It is widely accepted that AMPK phosphorylation at Thr^172^ and its subsequent activation plays a critical role in the exercise-induced glucose uptake. Activation of AMPK leads to GLUT4 translocation through phosphorylation of TBC1D1 at AMPK-dependent phosphorylation sites, including Ser^237^.^(19,20)^ Although p-AMPK levels in the basal state were comparable among groups, in response to exercise the levels were higher in the starch pair-fed and ISO *ad libitum* group than in starch *ad libitum* group, and the highest level was observed in the ISO *ad libitum* group (Fig. 7A). Total proteins of AMPK were not different among groups (1.00 ± 0.06, 0.92 ± 0.05, 0.93 ± 0.07 in Starch *ad libitum*, Starch pair-fed, ISO *ad libitum*, respectively). For analysis of p-TBC1D1, the immunoblot intensities were not normalized by intensities of total TBC1D1 because we could not obtain reliable results of total TBC1D1 (Fig. 7B). No difference in p-TBC1D1 level among groups was observed in the basal state (Fig. 7B), but immediately after exercise, the level in the ISO *ad libitum* group was higher than those in other groups (Fig. 7B).

**Fig. 8:**
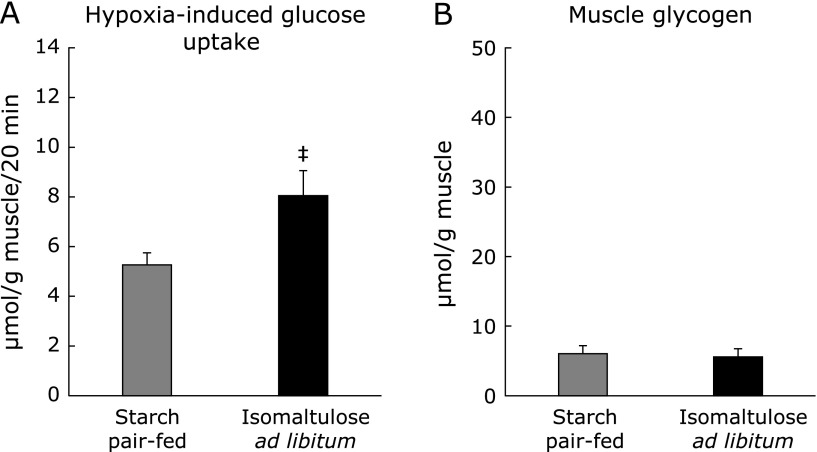
Hypoxia-induced glucose uptake. After 3-h fasting following the end of dark cycle, muscles were dissected out, and then were incubated for 80 min in hypoxygenated KHB under hypoxic condition. After that, muscles were further incubated for 20 min in oxygenated KHB. After the incubation, glucose uptake was measured in oxygenated KHB with 2-deoxyglucose (A). Some muscles were used for measurement of muscle glycogen content (B). Values are means ± SE (*n* = 6). ^‡^*p*<0.05 vs starch pair-fed group.

Although AMPK activation is an important component in the mechanism of exercise-induced glucose uptake, other insulin- and AMPK-independent signaling pathways or pathways that interact with AMPK have also been proposed to influence this process.^(20)^ In contrast to exercise stimulation, exercise-related hypoxia stimulation facilitates glucose uptake in a completely AMPK-dependent manner.^(21)^ Therefore, to obtain further evidence supporting the idea that promotion of exercise-induced glucose uptake by ISO feeding is due to higher activation of the AMPK signaling pathway, we examined the effect of ISO feeding on hypoxia-induced glucose uptake (Fig. 8A). In a comparison between the starch pair-fed group and ISO *ad libitum* group, ISO feeding resulted in higher glucose uptake in response to hypoxia. Glycogen level immediately after hypoxia did not differ between groups (Fig. 8B).

**Fig. 9:**
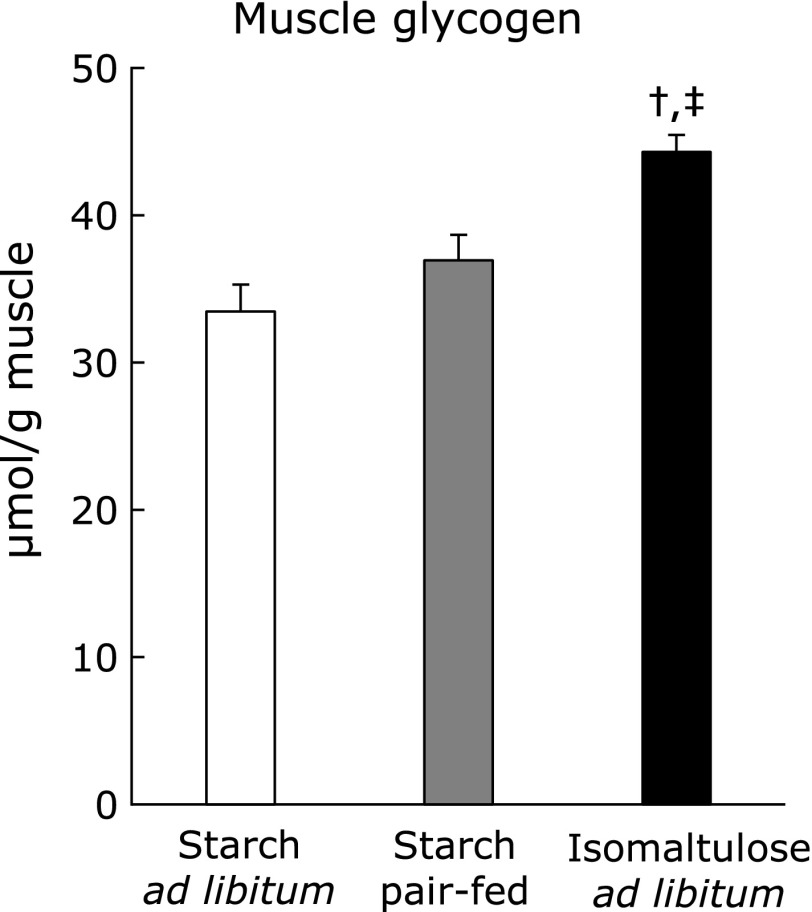
After 3-h fasting following the end of dark cycle, rats underwent swimming exercise. Immediately after the exercise and 2 h after the exercise, rats were received glucose solution orally to facilitate glycogen recovery. At 4 h after the exercise, muscles were dissected out, and were used for measurement of muscle glycogen content. Values are means ± SE (*n* = 6). ^†^*p*<0.05 vs starch *ad libitum* group. ^‡^*p*<0.05 vs starch pair-fed group.

Physical exercise promotes glucose uptake not only during exercise, but also during post-exercise states, and these effects are thought to contribute to glucose management in insulin-resistant subjects. In post-exercise states, glucose taken into skeletal muscle is mainly converted to glycogen in both insulin-dependent and -independent manners.^(22)^ Because our results demonstrated that ISO feeding facilitated glucose uptake in response to both insulin and exercise stimuli, we expected that ISO would have a positive impact on glycogen recovery after exercise. Indeed, as shown in Fig. 9, glycogen level 4 h after exercise was higher in the ISO *ad libitum* group than in the other groups.

## Discussion

The accumulated evidence shows that chronic ISO treatment holds promise for countering insulin-resistant metabolic disorders. The positive effect of chronic ISO treatment on regulation of glucose homeostasis can be attributed to multiple mechanisms. Two previous studies from the same group demonstrated that this regimen induces several favorable adaptations in various tissues,^(6,7)^ however, less attention has been paid to muscle adaptations. In this study, we demonstrated for the first time that ISO treatment significantly enhanced muscle insulin action in isolated skeletal muscle, and that this effect could be induced even by short-term replacement of starch with ISO, without reduction of visceral fat mass. This short-term effect is also likely to play an important role in the regulation of glucose metabolism in muscles chronically treated with ISO.

A bolus challenge of carbohydrate acutely stimulates secretion of incretin glucagon-like peptide (GLP) 1, and its secretion is amplified by ISO ingestion compared to sucrose.^(23)^ GLP1 functions in glucose-dependent insulin secretion, and also works on muscle glucose uptake,^(24,25)^ arterial vasorelaxation^(26)^ and recruitment of muscle microvasculature.^(27,28)^ Because of the presence of these *in vivo* factors, we elected to use an *ex vivo* system to reveal the molecular mechanism of the response of skeletal muscle to ISO treatment. Our results obtained in isolated skeletal muscle, showing comparable basal levels of glucose uptake among groups, strongly suggest that a direct effect of circulating GLP1 and a secondary effect mediated by hemodynamics can be ruled out as explanations for the results of this study.

Only one previous study has quantified the effect of ISO treatment on muscle-specific glucose uptake.^(9)^ In that report, the authors measured insulin-stimulated glucose uptake *in vivo* in soleus and gastrocnemius muscles from the rats treated ISO for 4 weeks, but observed no significant difference when the measurement was made following a 12-h fasting period after the end of treatment. Notably in this regard, a previous study by our group provided evidence that 24-h-lasting fasting amplified insulin-stimulated glucose uptake in comparison with the fed condition,^(13)^ demonstrating that short-term fasting has an insulin-sensitizing effect on skeletal muscle. As observed in ISO treatment, a lower level of circulating insulin is also commonly observed under fasting conditions. This similarity led us to consider the possibility that ISO would sensitize muscle insulin action through some mechanism shared with the insulin-sensitizing effect induced by fasting. The importance of the time when peripheral tissues are exposed to insulin in the regulation of insulin action has been suggested previously.^(29)^ Accordingly, the effect of ISO on muscle insulin action might tend to be masked when it is measured under fasting conditions.

What is the molecular mechanism of the insulin-sensitizing effect of ISO on muscle glucose uptake? The major determinant of the magnitude of muscle glucose uptake is GLUT4 protein content and GLUT4 translocation. Under many conditions, GLUT4 protein content is proportional to the insulin responsiveness of glucose uptake.^(30,31)^ Thus, fasting, by maintaining a lower circulating insulin level, stimulates an increase in GLUT4 protein content.^(13)^ In this study, we again observed that mild food restriction by pair-feeding of starch and coupled ISO feeding both increased GLUT4 protein content in comparison with starch feeding *ad libitum*. However, this increase is unlikely explain the difference in insulin-stimulated glucose uptake between the starch pair-fed and ISO *ad libitum* groups because the magnitude of GLUT4 protein content was comparable between the two groups.

Multiple studies have emphasized the important role of TBC1D4 in the process of GLUT4 translocation in response to insulin.^(19,20)^ In the basal state, the active Rab GTPase-activating protein (GAP) domain on TBC1D4 restrains exocytotic GLUT4 translocation. In the insulin-stimulated state, activated PI3K phosphorylates multiple Akt-phosphorylation sites, including Thr^642^ and Ser^588^. These sites are predicted to inhibit Rab-GAP activity, thereby relieving the inhibitory effect on GLUT4 translocation. In this study, the highest levels of TBC1D4 phosphorylation at these sites were observed in ISO-treated muscles, although the levels were not statistically different from those observed in starch pair-fed animals. These results indicate that up-regulation of TBC1D4 might be in with the enhanced action of insulin on glucose uptake after ISO treatment.

Besides insulin stimulation, exercise and exercise-related stimuli, such as muscle contraction and hypoxia,^(15,19–21)^ are well known to induce GLUT4 translocation. Although these stimuli facilitate glucose uptake through PI3K-independent signaling mechanisms, under some conditions when glucose uptake in response to insulin is elevated, glucose uptake induced by these stimuli is also amplified.^(11)^ In this study, we demonstrated for the first time that ISO treatment also amplified exercise-induced glucose uptake. Like TBC1D4, TBC1D1 has been predicted to play a role in exercise-induced GLUT4 translocation. Physical exercise and exercise-related stimuli increase phosphorylation at some sites in TBC1D1, including Ser^237^ in an AMPK-targeted motif, which potentially functions in AMPK-dependent GLUT4 translocation.^(19,20)^ Our results demonstrated that the level of exercise-induced phosphorylation TBC1D1 at Ser^237^ tended to be highest in ISO-treated muscles, with a significant difference relative to starch *ad libitum* feeding. Although effect of ISO on total TBC1D1 protein was obscure, regulation of TBC1D1 including its phosphorylation could amplify GLUT4 translocation, thereby contributing to enhancement of exercise-induced glucose uptake by ISO treatment. The role of TBC1D1 in regulation of insulin-stimulated glucose uptake is under debate.^(19,20)^ To obtain further insight into the action of ISO on skeletal muscle, future studies should investigate whether a common mechanism is responsible for amplification of glucose uptake by insulin and exercise/exercise-related stimuli. The measurement of GLUT4 translocation with response of related molecules against different stimuli would contribute the analysis of the mechanism.

AMPK is a master switch that regulates exercise-related metabolic responses/adaptations, and is also a therapeutic target for several metabolic disorders, including insulin-resistant obesity and diabetes.^(32)^ Another important observation in this study was that ISO treatment enhanced exercise-induced AMPK activation. These results are supported by our previous finding that glucose uptake by hypoxia, which is 100%-AMPK-dependent,^(21)^ was increased to the greatest extent by ISO *ad libitum* feeding. The increased AMPK response may, at least in part, explain the higher level of TBC1D1 phosphorylation in ISO-treated muscle. In contrast to the effect of exercise on AMPK, the basal phosphorylation level was not affected by ISO treatment, suggesting that ISO treatment alone does not induce significant AMPK-dependent beneficial effects on skeletal muscle. Therefore, ISO treatment together with AMPK-stimulating treatment, such as physical exercise, might further intensify the effects of ISO on muscle insulin action and glucose homeostasis in comparison with either treatment alone. Contrary to our results, a previous study reported that basal phosphorylation level of AMPK increased relative to control as a result of chronic (4-week) ISO feeding.^(9)^ Although we cannot explain the discrepancy between our results, possible reasons include differences in muscle fiber-type (fast fiber–dominant epitrochlearis vs slow fiber–dominant soleus muscle), duration of the experiments (12-h vs 4-week), duration of fasting (3-h, 12-h), or composition of the diet.

With regard to exercise, we observed an additional effect of ISO treatment on muscle glucose metabolism. Specifically, we obtained the first evidence demonstrating that ISO treatment affects muscle glycogen metabolism. In this study, glycogen levels were comparable between starch pair-feeding and ISO *ad libitum* before exercise, whereas glycogen recovery following glycogen-depleted exercise was enhanced. Because post-exercise glycogen recovery is regulated by both insulin-dependent and -independent glucose uptake,^(22)^ facilitation of these forms of glucose uptake by ISO treatment may explain the enhancement in glycogen recovery. Because impairment of glycogen synthesis is a feature of insulin-resistant subjects, an increase in glycogen synthesis following ISO treatment could exert beneficial effects on glucose management in such subjects. Also, muscle glycogen is well known to be a critical energy source during prolonged strenuous exercise, and its depletion is strongly linked to development of muscle fatigue.^(33)^ Therefore, ISO treatment represents a new nutritional approach to obtaining greater muscle glycogen in subjects who engage in competitive sports.

A previous study reported that chronic ISO treatment (2 months) significantly decreased liver TG content,^(8)^ although this is inconsistent with the results of other studies.^(7,9)^ Furthermore, no previous study has demonstrated an effect of ISO on muscle TG content. Surprisingly, our present results demonstrate that just 12 h of ISO treatment significantly decreased muscle TG content. This reduction in the level of negative regulator of insulin action could play a part in the enhancement of insulin action by ISO treatment. Although it remains unclear whether reduction to a level lower than normal could positively influence insulin action even in non–insulin-resistant muscles, if this phenomenon also occurs in TG-rich insulin-resistant muscle, it would represent an attractive feature of ISO. In this study, we could not reveal all of the functional mechanisms of ISO for the insulin-nonspecific effects on glucose uptake. Nevertheless, it is reasonable to hypothesize that the promotion by ISO of glucose uptake in response insulin and exercise might occur partially through the same mechanism that underlies the glucose transport system.

In conclusion, our results demonstrate that short-term replacement of starch with ISO enhanced insulin-dependent glucose uptake in isolated rat skeletal muscle. This insulin-sensitizing effect occurred independently of changes visceral fat mass, locomotor activity, and content of muscle glycogen and GLUT4 protein, but was associated with a reduction in muscle TG content. Furthermore, ISO treatment also amplified exercise-induced AMPK activation and glucose uptake. In addition to the chronic effects of ISO, transient substitution of carbohydrate with ISO, together with exercise, represents a potentially effective approach for the management of insulin resistance.

## Figures and Tables

**Table 1:** Composition of diets

(g/100 g)	Starch	Sucrose	Isomaltulose
Casein	25.2	25.2	25.2
Sucrose	0	56.8	0
α-Corn starch	56.8	0	0
Isomaltulose	0	0	56.8
Corn oil	5.3	5.3	5.3
Vitamine mix (AIN-76)	2.0	2.0	2.0
Mineral mix (AIN-76)	6.1	6.1	6.1
DL-Methionine	0.4	0.4	0.4
Cellulose	4.0	4.0	4.0
Choline bitartrate	0.2	0.2	0.2

**Table 2:** Blood parameters

	Starch *ad libitum*	Starch pair-fed	Isomaltulose *ad libitum*
Blood glucose (mg/dl)	105 ± 5	83 ± 4^†^	94 ± 3^‡^
Plasma insulin (ng/ml)	0.52 ± 0.08	0.26 ± 0	0.40 ± 0.13
Plasma free fatty acid (mEq/L)	0.52 ± 0.10	0.90 ± 0.04^†^	0.61 ± 0.04^‡^
Plasma glycerol (µg/ml)	9.28 ± 1.75	13.90 ± 1.05^†^	9.28 ± 1.7^‡^
Plasma triglyceride (mg/ml)	1.64 ± 0.28	0.95 ± 0.11^†^	0.27 ± 0.03^†,‡^

Values are means ± SE (*n* = 6–8). ^†^*p*<0.05 vs starch *ad libitum* group. ^‡^*p*<0.05 vs starch pair-fed group.

## Abbreviations

AMPK: AMP-activated protein kinase
BSA: bovine serum albumin
2DG: 2-deoxyglucose
2DG6P: 2-deoxyglucose-6-phosphate
FFA: free fatty acid
GAP: GTPase-activating protein
GI: glycemic index
GLP1: incretin glucagon-like peptide 1
GLUT4: glucose transporter 4
ISO: isomaltulose
KHB: Krebs-Henseleit buffer
PCA: perchloric acid
PI3K: phosphatidylinositol-3 kinase
TG: triglyceride

## References

[1] Augustin LS, Kendall CW, Jenkins DJ (2015). Glycemic index, glycemic load and glycemic response: An International Scientific Consensus Summit from the International Carbohydrate Quality Consortium (ICQC).. Nutr Metab Cardiovasc Dis.

[2] Maki KC, Phillips AK (2015). Dietary substitutions for refined carbohydrate that show promise for reducing risk of type 2 diabetes in men and women. J Nutr.

[3] Lina BA, Jonker D, Kozianowski G (2002). Isomaltulose (Palatinose): a review of biological and toxicological studies. Food Chem Toxicol.

[4] Atkinson FS, Foster-Powell K, Brand-Miller JC (2008). International tables of glycemic index and glycemic load values: 2008. Diabetes Care.

[5] Okuno M, Kim MK, Mizu M, Mori M, Mori H, Yamori Y (2010). Palatinose-blended sugar compared with sucrose: different effects on insulin sensitivity after 12 weeks supplementation in sedentary adults. Int J Food Sci Nutr.

[6] Matsuo K, Arai H, Muto K (2007). The anti-obesity effect of the palatinose-based formula inslow is likely due to an increase in the hepatic PPAR-α and adipocyte PPAR-γ gene expression. J Clin Biochem Nutr.

[7] Sato K, Arai H, Mizuno A (2007). Dietary palatinose and oleic acid ameliorate disorders of glucose and lipid metabolism in Zucker fatty rats. J Nutr.

[8] Arai H, Mizuno A, Matsuo K (2004). Effect of a novel palatinose-based liquid balanced formula (MHN-01) on glucose and liquid metabolism in male Sprague-Dawley rats after short- and long-term ingestion. Metabolism.

[9] Ohminami H, Amo K, Taketani Y (2014). Dietary combination of sucrose and linoleic acid causes skeletal muscle metabolic abnormalities in Zucker fatty rats through specific modification of fatty acid composition. J Clin Biochem Nutr.

[10] Oizumi T, Daimon M, Jimbu Y (2007). A palatinose-based balanced formula improves glucose tolerance, serum free fatty acid levels and body fat composition. Tohoku J Exp Med.

[11] Cartee GD, Holloszy JO (1990). Exercise increases susceptibility of muscle glucose transport to activation by various stimuli. Am J Physiol.

[12] Sano A, Koshinaka K, Abe N (2012). The effect of high-intensity intermittent swimming on post-exercise glycogen supercompensation in rat skeletal muscle. J Physiol Sci.

[13] Koshinaka K, Kawasaki E, Hokari F, Kawanaka K (2009). Effect of acute high-intensity intermittent swimming on post-exercise insulin responsiveness in epitrochlearis muscle of fed rats. Metabolism.

[14] Kawanaka K, Tabata I, Tanaka A, Higuchi M (1998). Effects of high intensity intermittent swimming on glucose transport in rat epitrochlearis muscle. J Appl Physiol (1985)..

[15] Cartee GD, Douen AG, Ramlal T, Klip A, Holloszy JO (1991). Stimulation of glucose transport in skeletal muscle by hypoxia. J Appl Physiol (1985)..

[16] Hansen PA, Gulve EA, Holloszy JO (1994). Suitability of 2-deoxyglucose for *in vitro* measurement of glucose transport activity in skeletal muscle. J Appl Physiol (1985)..

[17] Passonneau JV, Lauderdale VR (1974). A comparison of three methods of glycogen measurements in tissue. Anal Biochem.

[18] Passonneau JV, Lowry OH (1993). Enzymatic Analysis. A Practical Guide.

[19] Cartee GD (2015). Roles of TBC1D1 and TBC1D4 in insulin- and exercise-stimulated glucose transport of skeletal muscle. Diabetologia.

[20] Richter EA, Hargreaves M (2013). Exercise, GLUT4, and skeletal muscle glucose uptake. Physiol Rev.

[21] Mu J, Brozinick JT Jr, Valladares O, Bucan M, Birnbaum MJ (2001). A role for AMP-activated protein kinase in contraction- and hypoxia-regulated glucose transport in skeletal muscle. Mol Cell.

[22] Price TB, Rothman DL, Taylor R, Avison MJ, Shulman GI, Shulman RG (1994). Human muscle glycogen resynthesis after exercise: insulin-dependent and -independent phases. J Appl Physiol.

[23] Ang M, Linn T (2014). Comparison of the effects of slowly and rapidly absorbed carbohydrates on postprandial glucose metabolism in type 2 diabetes mellitus patients: a randomized trial. Am J Clin Nutr.

[24] González N, Acitores A, Sancho V, Valverde I, Villanueva-Peñacarrillo ML (2005). Effect of GLP-1 on glucose transport and its cell signalling in human myocytes. Regul Pept.

[25] Acitores A, González N, Sancho V (2005). Participation of protein kinases in the stimulant action of GLP-1 on 2-deoxy-D-glucose uptake by normal rat skeletal muscle. Horm Metab Res.

[26] Richter G, Feddersen O, Wagner U, Barth P, Göke R, Göke B (1993). GLP-1 stimulates secretion of macromolecules from airways and relaxes pulmonary artery. Am J Physiol.

[27] Subaran SC, Sauder MA, Chai W (2014). GLP-1 at physiological concentrations recruits skeletal and cardiac muscle microvasculature in healthy humans. Clin Sci (Lond)..

[28] Chai W, Dong Z, Wang N (2012). Glucagon-like peptide 1 recruits microvasculature and increases glucose use in muscle via a nitric oxide-dependent mechanism. Diabetes.

[29] Daly M (2003). Sugars, insulin sensitivity, and the postprandial state. Am J Clin Nutr.

[30] Ren JM, Semenkovich CF, Gulve EA, Gao J, Holloszy JO (1994). Exercise induces rapid increases in GLUT4 expression, glucose transport capacity, and insulin-stimulated glycogen storage in muscle. J Biol Chem.

[31] Henriksen EJ, Bourey RE, Rodnick KJ, Koranyi L, Permutt MA, Holloszy JO (1990). Glucose transporter protein content and glucose transport capacity in rat skeletal muscles. Am J physiol.

[32] Day EA, Ford RJ, Steinberg GR (2017). AMPK as a therapeutic target for treating metabolic diseases. Trends Endocrinol Metab.

[33] Bergström J, Hermansen L, Hultman E, Saltin B (1967). Diet, muscle glycogen and physical performance. Acta Physiol Scand.

